# Accelerated radiotherapy pathways: a systematic review on same-day planning and treatment in radiation oncology

**DOI:** 10.1016/j.ctro.2026.101145

**Published:** 2026-03-22

**Authors:** Astrid Heusel, Hubert Gabrys, Lotte Wilke, Sophie Perryck, Madalyne Day, Nicolaus Andratschke, Stephanie Tanadini-Lang, Matthias Guckenberger, Sebastian M. Christ

**Affiliations:** Department of Radiation Oncology, University Hospital and University of Zurich, Zurich, Switzerland

**Keywords:** Accelerated Pathways, Same-Day Radiotherapy, SRS, Single-fraction SBRT

## Abstract

**Introduction and background:**

Same-day radiotherapy, integrating consultation, simulation, planning, and treatment within a single day, is an emerging approach in radiation oncology. Advances in imaging integration, automation, and adaptive planning have enabled shorter timelines between consultation and treatment initiation, but prospective clinical evidence remains limited. This systematic review evaluates indications, feasibility, treatment accuracy, and patient satisfaction with same-day radiotherapy.

**Materials and methods:**

A systematic literature search was conducted in August 2025 using *PubMed*, *Embase*, and the *Cochrane Library* in accordance with *PRISMA 2020 guidelines*. Eligible studies included prospective or observational reports of radiotherapy for solid malignancies delivered on the same day as treatment planning.

**Results:**

Of 124 identified records, 13 studies met inclusion criteria. Most were prospective (n = 9), with four retrospective studies. Median study size was 39 patients, with a median ECOG performance status of 1. Treatment intent was predominantly palliative (54%), followed by curative (31%) and ablative (15%). Bone metastases were the most common indication; curative treatments included lung, prostate, brain, breast, and uveal tumors. Stereotactic radiotherapy (54%) and hypofractionated external-beam radiotherapy (38%) were most frequently used. Procedure times ranged from 25 to 470 min. Patient satisfaction, reported in three studies, was consistently good. Treatment accuracy was only assessed in three (23%) studies.

**Conclusion:**

Current evidence supports the feasibility of same-day radiotherapy, particularly in *palliative* settings, with favorable patient satisfaction and robust treatment accuracy, where reported. Larger prospective studies are required to standardize workflows and assess outcomes in *curative* settings, as well as health-system, financial, and legal implications.

## Highlights

• Same-day radiotherapy is feasible with high accuracy across mainly palliative settings.
• Workflow times vary widely, they are longest in MR-guided adaptive pathways.
• Patient satisfaction is consistently good; broader prospective data are needed.

## Graphical abstract

*Source*: This Graphical Abstract was produced with *OpenAI. ChatGPT (GPT-5.2)*. https://chat.openai.com/. Accessed 21.02.2026.

**Figure:**
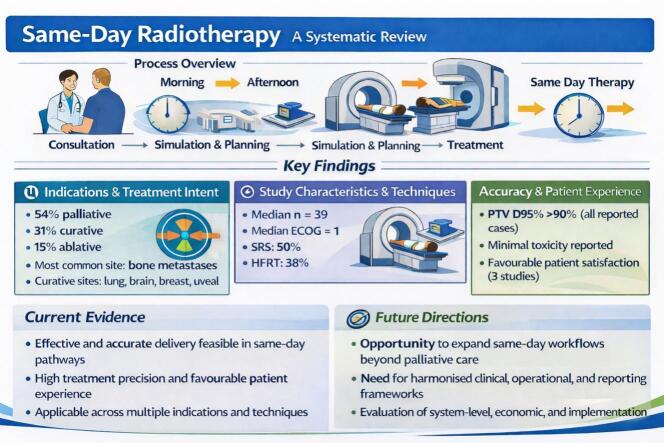

## Background and introduction

Radiotherapy is delivered through a highly structured, multidisciplinary pathways that typically involves multiple hospital visits and extended timelines [1]. After an initial consultation to confirm the indication and obtain informed consent, patients usually attend a separate appointment for treatment simulation, most often using computed tomography (CT) and, in selected cases, magnetic resonance imaging (MRI). When both modalities are required, examinations may occur on different days. Subsequent steps—including target delineation, treatment planning, quality assurance, and plan approval—extend the interval before the first treatment, often by several days [2].

In oncological *palliative* care, radiotherapy remains one of the most frequently used interventions, with well-established efficacy for pain relief and other symptom control, combined with generally favorable toxicity profiles [3]. However, particularly in this setting, long procedural timelines may compromise quality of life for patients with limited life expectancy [4]. As systemic therapies and local interventions continue to improve outcomes for patients with advanced and metastatic disease, the importance of durable symptom relief, convenience, and overall quality of life has become more pronounced [5].

Stereotactic body radiotherapy (SBRT) has emerged as a widely adopted local ablative treatment for small-volume disease across multiple anatomical sites, including lung, liver, prostate, pancreas, and spine [6]. SBRT achieves high local control rates, often exceeding 85–90% in early-stage tumors, with treatment delivered in a limited number of fractions [6]. In addition, single-fraction regimens have demonstrated comparable outcomes to multi-fraction schedules in selected tumor types and indications, with respect to toxicity, local control, progression-free survival, and overall survival, yet robust data for widespread adoption of single-fraction regimens is lacking [7]. These findings highlight opportunities to further streamline treatment delivery and suggest that highly conformal radiotherapy can be integrated into accelerated patient pathways without compromising efficacy.

Historically, same-day radiotherapy—combining simulation, planning, and treatment within a single day—was largely confined to emergencies such as spinal cord compression, great vessel obstruction, or tumor bleeding, typically delivered with three-dimensional conformal radiotherapy (3DCT) techniques [8]. More recently, advanced delivery methods such as intensity-modulated and arc-based radiotherapy (IMRT/VMAT) have enabled more conformal dose distributions and reduced toxicity, an important consideration as radiotherapy is increasingly combined with novel systemic agents whose toxicity profiles are incompletely characterized [8].

Recent technological advances have further expanded the feasibility of same-day radiotherapy beyond emergency use. Online adaptive radiotherapy, diagnostic CT-based pathways, artificial intelligence-driven automation, and accelerated dose calculation all contribute to faster planning and delivery [9]. MR-guided radiotherapy enables real-time plan adaptation according to daily anatomy and gated treatment delivery, while several groups have validated treatment planning directly from diagnostic imaging datasets [10], [11]. Collectively, these innovations create opportunities to substantially shorten the time from consultation to treatment initiation, reduce the travel and logistical burden for patients, and broaden access to advanced radiotherapy in both palliative and curative settings.

In this context, we conducted a systematic literature review to synthesize the current evidence on same-day planning and radiotherapy, with a focus on its indications, clinical feasibility, treatment accuracy, patient satisfaction, and procedural considerations such as pathway duration and consent procedure.

## Materials and methods

A systematic search of PubMed, Embase, and the Cochrane Library was conducted in August 2025 in accordance with the *Preferred Reporting Items for Systematic Reviews and Meta-Analyses (PRISMA) 2020* guidelines. The review protocol was prospectively registered on the *PROSPERO* database; the *PROSPERO* registration number is CRD420251116160.

The search strategy was: ((same-day) OR (one day) OR (simulation-free) OR (single-visit) OR (rapid-access) OR (on-table adaptive) OR (direct to unit)) AND (radiotherapy[MeSH Terms]). Eligible studies included prospective or observational investigations that evaluated same-day treatment planning and initiation of radiotherapy in patients with solid malignancies. Publications between 2000 and 2025 were considered. Case reports and *in-silico studies* were excluded from the analysis.

**Fig. 1:**
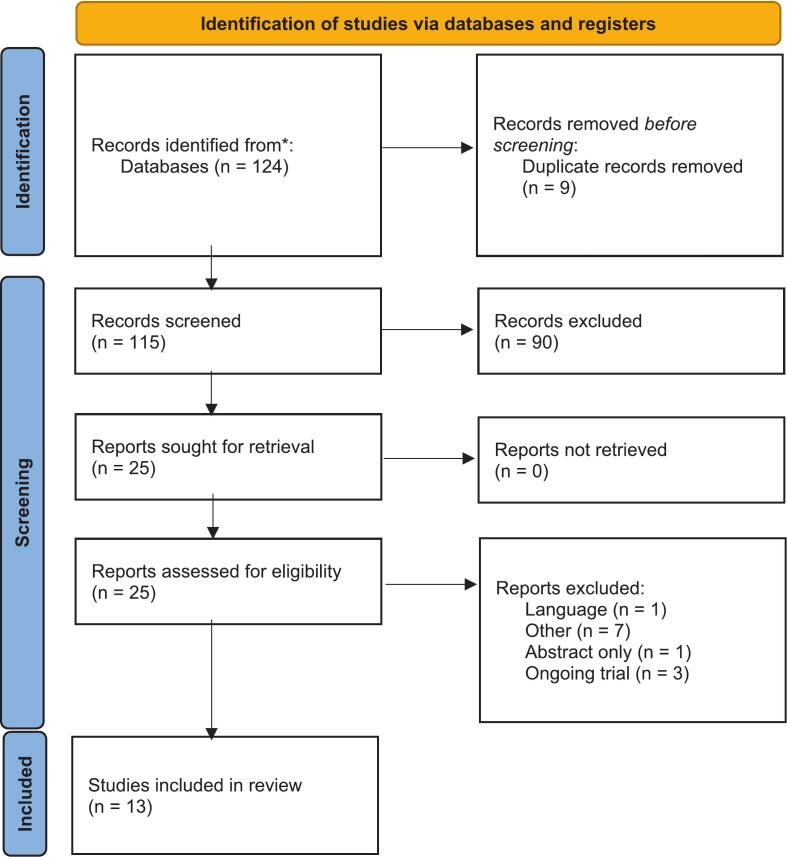
Identification of studies according to the PRISMA guidelines 2020.

Study selection and data extraction were performed by a single reviewer (AH) and independently verified by a second reviewer (SMC). In total, 124 (100%) records were identified, of which 9 (7%) were removed as duplicates. The remaining 115 (93%) records were screened, with 25 (20%) assessed for eligibility. Twelve studies (10%) were excluded, resulting in 13 (10%) studies being included in the final analysis (Fig. 1, CONSORT diagram).

Subsequent data extraction was conducted manually and included study characteristics (year of publication, study design, primary and secondary endpoints), patient demographics (sample size, median age, Eastern Cooperative Oncology Group (ECOG) performance status, primary tumor histology, treatment site), treatment details (dose and fractionation, planning approach, delivery technique, procedure times), and feasibility as well as patient-related outcomes (patient satisfaction, toxicity).

## Results

Of the thirteen studies which met the inclusion criteria, nine (75%) were prospective trials. Median size in terms of study participants was 39 patients (range: 10–96), with a median ECOG performance score of 1 (range: 0–4). The predominant treatment indication was palliation (7 studies; 54%), followed by curative intent treatments (4 studies; 31%), and local ablative interventions (2 studies; 15%) *(*Table 1*)*. The most frequently treated sites were bone metastases (spine and non-spine), followed by the abdomen and pelvis. In studies with curative intent, treatment sites included lung, prostate, central nervous system, breast and uvea. The most common primaries were lung cancer (small and non– small lung cancer) (16%), breast cancer (14%), skin cancer (melanotic and non-melanotic) (14%), prostate cancer (14%) and other urological cancers (11%).

**Table 1:** Basic study characteristics.

**Type of study**	**n**	**(%)**
- prospective	10	77
- retrospective	3	23
**Treatment intent**		
- palliative	7	54
- curative	4	31
- local ablation	2	15
**Site of treatment**		
- Bone	3	23
- CNS	2	15
- Lung	1	8
- Prostate	1	8
- Uvea	1	8
- Breast	1	8
- Multi- site*	4	30
**Primary malignancy**		
- Lung (NSCLC/ SCLC)	7	16
- Breast	6	14
- Skin (melanotic/ non melanotic)	6	14
- Prostate	6	14
- Urological (other than prostate)	5	11
- Gastrointestinal	3	7
- Primary brain tumor	2	4
- Head & neck	2	4
- Hematological	1	2
- Gynecological	1	2
- Bone and soft tissue	1	3
- Not otherwise classified	4	9
**Number of patients (median, range)**	39 (10–96)	
**Study end point**		
- Single end point	5	38
- Multiple end points	8	62
**End points**		
- Feasibility	11	34
- Procedure time	6	18
- Patient satisfaction	4	13
- Efficacy	4	13
- Toxicity	3	9
- Dosimetric accuracy	4	13

*Notes:* CNS = Central nervous system; NSCLC = Non-small-cell lung cancer; SCLC = Small-cell lung cancer.

* Bone, brain, abdomen/ pelvis.

Most studies had multiple endpoints (8 studies; 62%). The majority of studies addressed feasibility (11 studies; 84%), followed by procedure time (6 studies; 46%), patient satisfaction (4 studies; 33%), as well as treatment efficacy (4 studies; 31%). Four studies reported on toxicity and dosimetric accuracy.

Before treatment day, a telephone consultation with a radiation oncologist was undertaken in two studies only; one of these also mentioned having scheduled a second consultation on the treatment day, to obtain consent from the patient prior to arrival in the department *(*Table 2*)*. Informed consent was obtained on the treatment day itself in three studies (25%), while three studies (25%) reported on prior in-house consultations with a radiation oncologist.

**Table 2:** Treatment planning.

**Informed consent**	n	(%)
- Prior in-house consultation	3	25
- Same day consultation	3	25
**Dummy treatment plan**	4	31
**Imaging for dose calculation**		
- Planning CT	5	38
- kV-CT	3	23
- MRidian® pathway	3	23
- diagnostic CT - PSMA-PET/CT	1 1	8 8

*Notes:* CT = Computed tomography.

Treatment simulation was performed using computer tomography (CT) simulation in five studies (38%) and magnetic resonance (MR) simulation in three studies *(*Table 2*)*. For dose calculation, the following imaging modalities were applied: planning CT (5 studies; 38%), MR-only techniques, using the MRIdian® system (3 studies; 23%), and kV-CT (3 studies; 23%). One study reported dose calculations based on a diagnostic CT and an other one based on PSMA-PET/CT. A dummy treatment plan was calculated before the treatment day in four studies (31%), using previously acquired diagnostic CT or PSMA-PET/CT scans. The dummy treatment plans were subsequently recalculated at the time of simulation using either an MR-only pathway or kV-CT.

As detailed in Table 3, the predominant treatment modalities were stereotactic radiotherapy (7 studies; 54%) and hypofractionated radiotherapy (5 studies; 38%). Stereotactic radiotherapy was applied for both palliative purposes (bone and brain metastases) and curative intents (uveal melanoma, primary cystic brain tumors, and lung tumors), with prescribed total doses ranging from 12 Gy to 36.25 Gy. The most common hypofractionation regimens (1 × 8 Gy, 5 × 4 Gy, and 10 × 3 Gy) were exclusively used in studies with palliative goals. Only one study employed a normofractionated regimen (50 Gy in 25 fractions or 42.4 Gy in 16 fractions) for adjuvant radiotherapy in breast-conserving treatment settings.

**Table 3:** Treatment delivery and patient related outcomes.

**Treatment modality**	**n**	**(%)**
- Stereotactic RT (SRS, SRT, SBRT)	7	54
- Hypofractionated EBRT - Normofractionated EBRT	5 1	38 8
**Dose and fractionation**		
- Total dose stereotactic RT	12–36.25 Gy	
- Total dose hypofractionated EBRT - Total dose normofractionated EBRT	8–37.5 Gy 42.4–50 Gy	
**Toxicity CTCAE**	**n**	**(%)**
- Toxicity assessment	4	31
- ≥ 3	1	25
**Patient satisfaction**		
- Good	3	23

Patient satisfaction was reported in three studies (23%) and was generally characterized as “good”. Toxicity was assessed in four studies (31%), with one study reporting using the *Common Terminology Criteria for Adverse Events (CTCAE)* toxicity scale.

Data on procedural times were not reported uniformly across the nine included studies *(*Table 4*)*. Only one study provided information on the consultation duration. Treatment simulation was described in three studies, with simulation times ranging from 2.8 to 66 min; in the two studies with MR-guided simulation, simulation times ranged from 21 to 66 min, whereas the CBCT-based study reported a simulation time ranging from 2.8 to 25.6 min. Target delineation, which includes the delineation of organs at risk and treatment volumes, was reported in only three studies; one of these studies used a semi-automated segmentation tool. Similarly, planning and plan approval were described in three studies, with durations of 0.9–176 min and 2.8–81 min, respectively. The overall total time or *time in center* representing the cumulative procedure time, was reported in five studies (38%) and ranged from 25 to 470 min. Two of these studies featured an MR-guided technique with a median total of 130–396 min.

**Table 4:** Procedure times.

**Study**	**Consultation**	**Simulation**	**RT planning**					**Positioning**	**RT treatment**	**Total time**
Lee et al. 2015			***Target delineation***		***Plan calculation***	***Plan approval***	***Quality assurance***			
			23 (5–62)		56 (21–176)	23 (3–81)	33 (9–114)			
		***CT simulation to RT treatment***								
		166 (92–244)								
Wilson et al. 2019			***Time from CT to completion of RT treatment***							
			378 (180–570)							
Nelissen et al. 2022		***Patient setup + CBCT***	***Influencer generation***	***Target + influencer generation + approval***	***Plan calculation***	***Plan approval***		***RT treatment + CBCT + off-couch***		85 (50–130)
		8.5 (2.8–25.6)	1.2 (0.9–5.0)	2.2 (0.3–23.2)	1.4 (0.9–7.1)	4.3 (2.8–6.2)		8.1 (4.0–19.2)		
Palacios et al. 2022	18	66	168						72	396
Schiff et al. 2023			***From simulation to start of first treatment***							
		33 (21–63)	407 (306–542)					8 (4–28)	22 (11–106)	
De Leon et al. 2024			***Image fusion + contouring (Sim- free)***		***Treatment planning (Sim- free)***					130
			34		97					
O'Neil et al. 2024			***Time in center (CTsim workflow)***							
			282							
			***Time in center (dCT workflow)***							
			25							
Christ et al. 2025		***On table time***								
		65 (57–112)								
Mitsuhashi et al. 2025		***End of consultation to end of CT simulation***	***From end of simulation to end of treatment***							225 (85–470)
		52 (10–345)	35 (10–345)							

With respective to treatment accuracy, three studies reported on the planning target volume (PTV) coverage, with the D95% exceeding 90% (range: 90–100) in all cases *(*Table 5*)*. Importantly, all of these studies were conducted in the *palliative* treatment setting. Additionally, some studies reported the use of Monte Carlo–based dose recalculations as part of patient-specific quality assurance, with plan verification performed using gamma index analysis applying distance-to-agreement and dose-difference criteria of 2 mm/2%, 2 mm/3%, and 3 mm/3%, respectively. Some other studies described compliance to the center specific protocols and standard operating procedures, without including further details. Of note, only one study conducted with *curative* treatment intent evaluated the differences in dosimetric parameters between simulation-free plans and recalculations based on each patient’s individual relative electron density (RED). The mean difference between the patients’ actual RED values and the population-based RED values for target volumes and organs at risk was 0.1%. When the recalculated plans were compared with the simulation-free plans, the average PTV D95% and GTV D98% were marginally lower, by 0.5% and 0.6%, respectively. Similarly, no clinically relevant differences were observed in the OAR parameters.

**Table 5:** Dose coverage.

***Study***	***PTV D95%***
Wilson et al. 2019	> 90%
Nelissen et al. 2022	94.6- >95%
Schiff et al. 2013	99.87% (92.2–100)

## Discussion

This systematic review synthesizes the available evidence on same-day radiotherapy pathways across both *palliative* and *curative* contexts. The literature search was specifically designed to identify studies describing workflows in which imaging, treatment planning, and radiotherapy delivery were completed within a single day. This approach was intentionally adopted to capture pathways that incorporated a defined planning process within a single-day timeline, rather than to encompass the full spectrum of accelerated or simplified radiotherapy concepts. While this focused methodology was intended to enhance conceptual consistency across included studies, it may have contributed to the relatively limited number of eligible publications.

Same-day radiotherapy comprises a heterogeneous spectrum of workflows that vary considerably in terms of technical complexity, resource requirements, clinical risk, and scalability. Of the 13 included studies into this systematic review, the majority were prospective (77%), albeit with modest median sample sizes (39 patients), reflecting the early and exploratory nature of this field. Patient performance status was generally favorable (median ECOG 1), which likely facilitated the feasibility of expedited pathways. Treatment intent was predominantly palliative (54%), but a substantial proportion of studies addressed curative (31%) and ablative (15%) indications, underscoring the growing interest in expanding same-day approaches beyond urgent or end-of-life settings.

The most common clinical scenarios for same-day radiotherapy were bone metastases, consistent with the established role of radiotherapy in palliation of skeletal pain [3]. Curative-intent pathways were reported in lung, prostate, central nervous system, breast, and uveal melanoma, suggesting that the concept is adaptable to various disease settings. Treatment techniques were largely advanced, with stereotactic radiotherapy (54%) and hypofractionation (38%) being the most frequently employed. This pattern aligns with broader trends in radiation oncology towards hypofractionation, where single- or few-fraction schedules naturally lend themselves to expedited care pathways [12]. The clinical rationale for same-day radiotherapy is most evident in the context of single-fraction or ultra-hypofractionated treatments, in which treatment planning and delivery can be completed within a single patient encounter. In contrast, the benefit of a same-day pathway for multi-fraction regimens is less immediately apparent and merits further consideration. Although the total treatment course is delivered over multiple sessions, the interval between consultation, simulation, treatment planning, and initiation of therapy represents a critical component of the overall care pathway. Same-day radiotherapy approaches may confer advantages by shortening the time to treatment initiation. In addition, reducing pre-treatment delays may help alleviate patient anxiety, streamline clinical workflows, and decrease the number of hospital visits required prior to the start of therapy, thereby enhancing patient convenience and optimizing resource utilization. Nevertheless, further investigation is required to better define the potential clinical benefits of this approach in multi-fraction settings, with particular emphasis on its impact on treatment efficiency and overall care delivery.

Procedure times varied substantially across studies, with reported totals ranging from 25 to 470 min. This wide variation reflects differences in treatment pathway design, imaging modality, and reporting methods. *Nelissen et al. (2023)* reported a median time of 85 min (range, 50–130) using diagnostic CT-based planning for 47 patients with bone metastases [13], whereas *O’Neil et al.* (2024) in their DART trial observed a large difference in *time in center*: 25 min for diagnostic CT-based pathways versus 282 min for CT simulation-based pathways [14]. *Mitsuhashi et al. (2025)* reported a median total procedure time of 225 min (range 85–470) for 96 patients, further illustrating variability in pathway times [15]. In the reviewed studies, MR-guided therapy pathways were consistently associated with longer times: *Palacios et al. (2022)* reported a median total time of 396 min for curative-intent single-fraction MR-guided lung SBRT [16], while *Schiff et al. (2022)* achieved a median duration of 407 min (range, 306–542) for palliative MR-guided treatments. These prolonged times reflect the complexity of MR-guided pathways, including gated delivery and adaptive replanning [17]. By contrast, *Christ et al. (2025)* reported shorter on-table times (median 65 min; range, 57–112) for MR-guided bone metastasis treatment, suggesting that patient pathway optimization can reduce time burdens [18]. Nevertheless, direct comparison across studies remains difficult due to the heterogeneity in fractionation schemes, imaging approaches, and definitions of procedure time.

Accuracy of treatment delivery was uniformly high, though not consistently reported. PTV coverage exceeded 90% in all studies that provided dosimetric data. Notably, these studies were conducted in the *palliative* setting, with *Schiff et al. (2022)* reporting near-complete coverage (median PTV-D95% 99.9%) [17]. Adaptive pathways demonstrated measurable advantages: *Nelissen et al. (2023)* documented significant improvements in CTV and PTV V95% with adapted versus scheduled plans (p < 0.005), highlighting the potential of same-day adaptive radiotherapy to enhance conformity and robustness despite anatomical changes [13]. Dosimetric accuracy was also evaluated by De Leon et al. (2024), who compared patient-specific RED values with population-based RED values for target volumes and organs at risk. The differences were minimal, averaging 0.1%. Furthermore, comparisons between recalculated plans and simulation-free plans revealed no significant discrepancies: the mean PTV D95% and GTV D98% were only marginally lower, by 0.5% and 0.6%, respectively. Likewise, no clinically relevant differences were observed in the parameters of organs at risk. These findings suggest that streamlined pathways may achieve dosimetric outcomes comparable to conventional approaches; however, the limited and heterogeneous nature of the available data precludes definitive conclusions regarding potential improvements in dosimetric quality. Still only a limited number of the included studies reported quantitative dosimetric outcomes, and these were predominantly confined to DVH-based metrics. Such parameters alone are insufficient to provide a comprehensive evaluation of treatment accuracy, particularly in stereotactic or adaptive contexts, where additional considerations—such as organ-at-risk dosimetry, plan robustness, geometric uncertainties, and direct comparisons with conventional workflows—are integral to a complete assessment.

Dosimetric uncertainties associated with same-day radiotherapy workflows – particularly those based on kV-CT planning in non-standard contexts or MR-only planning strategies – constitute an important methodological consideration when interpreting reported clinical outcomes. Although several of the included studies describe encouraging results, the dosimetric accuracy of these streamlined approaches is frequently derived from technical feasibility assessments or limited validation cohorts, rather than from direct, prospective comparisons with conventional CT-based workflows. Techniques such as MR-only planning, synthetic CT generation, or treatment planning on diagnostic imaging may introduce uncertainties related to electron density assignment, geometric distortion, image registration, and patient positioning. While the available literature generally demonstrates acceptable levels of dosimetric agreement, the significance of these differences and their clinical relevance remain insufficiently characterized in prospective clinical settings. Future investigations should therefore incorporate comparative study designs that systematically assess dosimetric endpoints and correlate them with clinical outcomes. Such work would be essential to determine whether the clinical results observed in same-day radiotherapy workflows are truly comparable to those achieved with standard pathways, or whether subtle dosimetric variations translate into differences in local control, toxicity, or other clinically meaningful endpoints.

Beyond technical feasibility, clinical and health-system implications must be considered. While several studies demonstrate the technical feasibility of completing same-day workflows, particularly in MR-guided settings, the reported procedure durations – occasionally approaching 6–7 h – may be challenging to integrate into routine clinical environments. Such extended treatment sessions are likely to impose substantial demands on staffing, equipment availability, and scheduling, and may therefore be difficult to reconcile with the requirements of standard clinical routine. In contrast, broader considerations related to organizational feasibility, resource allocation, staffing models, patient flow, and scalability to routine clinical practice remain only sparsely addressed in the current literature.

Patient-reported outcomes were not assessed consistently, but were favorable where available. Three studies reported on patient satisfaction, consistently described as “*good*”, and toxicity outcomes, though measured in only 33% of studies, were generally low and manageable. This is particularly relevant in palliative settings, where reducing treatment burden and maintaining quality of life are central goals. Future studies should more systematically assess patient satisfaction, qualify of life, and patient-reported outcomes (PROMs) with validated tools. One may postulate, that for patients, especially those with advanced disease, same-day radiotherapy reduces the burden of repeated hospital visits and associated travel. Even in curative settings, it may increase accessibility for patients in remote regions or with limited resources. At the departmental level, shorter care pathways can reduce the need for multiple appointments, improve scheduling flexibility, and optimize resource use, though the extended procedure times, especially of MR-guided pathways, may at least partially counteract some efficiency gains. Integration of automation, artificial intelligence-driven contouring, and adaptive planning will be pivotal to realize throughput advantages without compromising accuracy.

Last, but not least, legal and financial aspects need to be taken into consideration. Informed consent is a critical, legally sensitive step in radiation therapy administration, which must be carefully managed in expedited pathways. Studies varied widely: some obtained consent on the treatment day, others via prior in-house or telephone consultations. To ensure compliance with national ethical and legal standards, robust documentation and clear patient information are essential, particularly when consent is obtained remotely or under time constraints. Financially, many reimbursement systems remain structured around conventional multi-visit pathways. Same-day approaches, though potentially reducing overall system costs, may not be adequately covered under current funding models. Dialogue with payers and regulators will therefore be necessary to adapt reimbursement schemes to support innovation in care pathway designs, which center around the patient, rather than the healthcare system.

The implementation of same-day radiotherapy should integrate patient-, disease-, and workflow-related factors to support balanced clinical decision-making and safe implementation. Appropriate patient selection is central to same-day RT pathways. Key considerations include clinical urgency, the need to adhere to tight systemic therapy timelines. Patient-related factors should also be assessed, including performance status, adequacy of pain control, psychological aspects such as anxiety or claustrophobia, particularly in MR-guided settings, and the ability to tolerate immobilization, prolonged imaging, or extended in-room procedure times. From a clinical perspective, the rationale for same-day RT is strongest in single-fraction or very short-course regimens. Time-sensitive scenarios, such as bleeding, severe pain, or impending functional compromise, may also benefit from accelerated pathways. In addition, patients with substantial access barriers, such as frailty, long travel distances, or limited caregiver support, may also benefit from a reduction in pre-treatment visits. The principal anticipated advantage of same-day RT is a shorter time to treatment initiation, along with fewer hospital visits before the first fraction, improved patient convenience, and potentially better adherence. From an organizational standpoint, streamlined pathways may also reduce scheduling complexity and improve workflow efficiency. However, same-day RT workflows introduce several risks and limitations. Dosimetric uncertainties may arise when planning is performed on non-standard imaging datasets, such as diagnostic CT, kV-CT in treatment position, or MR-only workflows requiring synthetic CT generation. These approaches may be associated with uncertainties in electron density assignment, image registration, and geometric accuracy. In addition, condensed workflows may accentuate geometric uncertainties related to setup, deformation, or motion, and may limit opportunities for contouring review, peer review, and comprehensive quality assurance. Furthermore, certain same-day approaches, particularly MR-guided adaptive workflows, may involve prolonged in-room procedure times, which can challenge patient tolerance and impose substantial demands on staffing, equipment availability, and scheduling. Overall, same-day RT appears most appropriate for carefully selected patients with time-sensitive indications, short-course regimens, or significant logistical barriers to care. The potential benefits of reduced time to treatment and improved convenience should be balanced against dosimetric, geometric, and organizational risks, and further evidence is needed to support broader implementation.

In conclusion, the available evidence synthesized in this systematic review demonstrates that same-day planning and radiotherapy is both technically feasible and clinically acceptable, particularly in *palliative* radio-oncological care, with emerging evidence supporting the use in selected *curative* contexts. However, the heterogeneity of reporting, small sample sizes, and predominance of feasibility endpoints limit the strength of conclusions and its generalizability. Future research should aim for standardisation of patient pathway reporting, include patient-centred and economic outcomes, and evaluate legal and consent models appropriate for expedited care. Larger prospective studies and health-economic analyses may help to demonstrate not only feasibility but also equivalence in efficacy, safety, and financial sustainability compared with more conventional pathways. Ultimately, same-day radiotherapy has the potential to enhance patient convenience, preserve quality of life, and improve system efficiency, but successful implementation requires coordinated and multi-professionally integrated efforts across clinical, technical, financial, and legal domains.

## CRediT authorship contribution statement

**Astrid Heusel:** Conceptualization, Methodology, Formal analysis, Data curation, Writing – original draft. **Hubert Gabrys:** Methodology, Data curation, Writing – review & editing. **Lotte Wilke:** Writing – review & editing. **Sophie Perryck:** Writing – review & editing. **Madalyne Day:** Writing – review & editing. **Nicolaus Andratschke:** Supervision, Writing – review & editing. **Stephanie Tanadini-Lang:** Supervision, Writing – review & editing. **Matthias Guckenberger:** Supervision, Writing – review & editing. **Sebastian M. Christ:** Conceptualization, Methodology, Supervision, Writing – original draft, Writing – review & editing.

## Declaration of Competing Interest

The authors declare that they have no known competing financial interests or personal relationships that could have appeared to influence the work reported in this paper.
